# Impaired awareness of action-outcome contingency and causality during healthy ageing and following ventromedial prefrontal cortex lesions

**DOI:** 10.1016/j.neuropsychologia.2018.01.021

**Published:** 2019-05

**Authors:** Claire O’Callaghan, Matilde M. Vaghi, Berit Brummerloh, Rudolf N. Cardinal, Trevor W. Robbins

**Affiliations:** a Department of Psychiatry, University of Cambridge, Cambridge, UK; b Behavioural and Clinical Neuroscience Institute, University of Cambridge, Cambridge, UK; c Brain and Mind Centre, University of Sydney, Sydney, Australia; d Department of Psychology, University of Cambridge, Cambridge, UK; e MRC Cognition and Brain Sciences Unit, University of Cambridge, Cambridge, UK; f Department of Experimental Psychology and Methods, Leipzig University, Leipzig, Germany; g Liaison Psychiatry Service, Cambridgeshire and Peterborough NHS Foundation Trust, UK

**Keywords:** Contingency learning, Causality, Goal-directed behaviour, Awareness, Metacognition, Ventromedial prefrontal cortex lesion, Healthy ageing

## Abstract

Detecting causal relationships between actions and their outcomes is fundamental to guiding goal-directed behaviour. The ventromedial prefrontal cortex (vmPFC) has been extensively implicated in computing these environmental contingencies, via animal lesion models and human neuroimaging. However, whether the vmPFC is critical for contingency learning, and whether it can occur without subjective awareness of those contingencies, has not been established. To address this, we measured response adaption to contingency and subjective awareness of action-outcome relationships in individuals with vmPFC lesions and healthy elderly subjects. We showed that in both vmPFC damage and ageing, successful behavioural adaptation to variations in action-outcome contingencies was maintained, but subjective awareness of these contingencies was reduced. These results highlight two contexts where performance and awareness have been dissociated, and show that learning response-outcome contingencies to guide behaviour can occur without subjective awareness. Preserved responding in the vmPFC group suggests that this region is not critical for computing action-outcome contingencies to guide behaviour. In contrast, our findings highlight a critical role for the vmPFC in supporting awareness, or metacognitive ability, during learning. We further advance the hypothesis that responding to changing environmental contingencies, whilst simultaneously maintaining conscious awareness of those statistical regularities, is a form of dual-tasking that is impaired in ageing due to reduced prefrontal function.

## Highlights

• In both vmPFC lesions and ageing, intact responding to variations in action-outcome contingencies was maintained.
• In contrast, vmPFC damage and ageing impaired subjective awareness of action-outcome contingencies.
• vmPFC is not critical for contingency learning.
• vmPFC has a critical role in supporting awareness (metacognition) during learning.
• Impaired causal awareness in ageing may result from dual-tasking costs.

## 1 Introduction

The ability to detect causal relationships between actions and their outcomes is fundamental to adaptive behaviour. Humans and animals learn which events in the environment are direct consequences of their actions, versus those arising independently. An optimal behavioural strategy is to pursue those actions with a high likelihood of producing a reinforcing outcome, and avoid those that have no causal influence (Balleine and O'Doherty, 2010). Goal-directed behaviour, therefore, is not exclusively driven by the value attached to action-outcome associations; it also requires computation of the likelihood that an outcome will occur in the presence or absence of a given action (Baum, 1973, Dickinson and Balleine, 1994, Schultz, 2015).

This casual relationship is formalised as contingency (Rescorla, 1967). Contingency describes the difference between two probabilities: the contingent probability (P) of an outcome (O) given an action (A), i.e., [P(O|A)], and the non-contingent probability of that outcome occurring in the absence of that action [P(O|~A)] (Hammond, 1980). The difference between the two probabilities, denoted by ΔP, is expressed as ΔP = P(O|A)–P(O|~A). This probability expresses the overall strength of the action-outcome relationship (Liljeholm et al., 2011).

Regions of the ventromedial prefrontal cortex (PFC) are implicated in the behavioural expression of learned contingencies. Rats with lesions to the prelimbic cortex of the medial PFC show reduced sensitivity to contingency degradation (Balleine and Dickinson, 1998, Corbit and Balleine, 2003). Contingency can be degraded when the non-contingent probability of an action is increased, while its contingent probability is held constant. The agent is then faced with the occurrence of reinforcing outcomes that are not caused by their instrumental action. The adaptive strategy in this scenario is for the agent to reduce responding, reflecting their diminished causal influence on outcome delivery. Insensitivity to contingency degradation has also been shown in marmosets with lesions to the perigenual anterior cingulate cortex (area 32; a possible homologue of the rodent prelimbic cortex) and lateral orbitofrontal cortex (areas 11 and 13) (Jackson et al., 2016). These findings confirm a crucial causal role for PFC regions in the adaptation to environmental contingencies.

In humans undergoing functional magnetic resonance imaging whilst performing a contingency learning task, activation in the medial PFC, orbitofrontal cortex, and dorsomedial striatum (anterior medial caudate nucleus) increases as a function of response-outcome contingencies (Tanaka et al., 2008). Activation in the ventromedial PFC and anterior caudate nucleus, in particular, appears to track changes in the strength of the response-contingent relationship P(O|A) (Tanaka et al., 2008, Liljeholm et al., 2011). In contrast, activity in the inferior frontal gyrus and posterior caudate nucleus is found to scale with variations in non-contingent reward probabilities (P(O|~A)) (Liljeholm et al., 2011). These findings highlight the extent to which prefrontal brain regions supporting contingency computations are conserved across species; they also raise the possibility of specialisation in the neuronal circuitry underpinning contingent versus non-contingent information processing in humans.

An important extension achieved in studies of human contingency learning is the measurement of subjective awareness of causality. Unlike animal work, in which contingency learning is necessarily measured by response output, human participants can also report beliefs about the causal impact of their actions. This is achieved by having participants rate the extent to which their action caused reinforcer delivery, following each block of learning trials in which contingencies were systematically manipulated. This design permits a distinction between performance and awareness, capitalising on the unique advantage gained when animal methodologies are applied in the human arena (Weiskrantz, 2004). In healthy young adults, subjective causal ratings are sensitive to variations in contingent and non-contingent action-outcome associations (Chatlosh et al., 1985, Shanks and Dickinson, 1991, Wasserman et al., 1993, Tanaka et al., 2008, Liljeholm et al., 2011). Performance and awareness show comparable sensitivity, and they are strongly correlated. Regions of the medial PFC implicated in tracking response-outcome contingencies are also associated with subjective causal ratings (Tanaka et al., 2008). In healthy older subjects, however, despite responding appropriately to contingency changes, subjective detection of causality is mildly but significantly impaired for negative contingencies, where non-contingent outcomes occur more frequently than contingent outcomes (Mutter and Williams, 2004). Thus, in ageing, a behavioural dissociation appears between response output and subjective awareness.

Here, we aimed to explore whether the vmPFC, which has been linked to contingency learning in humans via correlational functional neuroimaging studies, is causally related to this process. To achieve this, we recruited individuals with vmPFC lesions, and a separate lesion control group with damage extrinsic to the vmPFC. We tested whether the vmPFC is required for both efficient response adaptation to contingency and for subjective awareness of causality. We predicted that performance and awareness would dissociate in the vmPFC group. To further distinguish between the impact of vmPFC lesion damage and ageing, we recruited a separate cohort of younger and older adults to investigate contingency learning across the lifespan. We anticipated that older adults would be impaired in their subjective ratings, despite maintaining intact response output. We predicted that by altering P(O|A) and P(O|~A) separately, specific deficits in processing contingent vs. non-contingent conditional probabilities would emerge in vmPFC lesions and ageing, respectively.

## 2 Methods and materials

### 2.1 Participants

#### 2.1.1 Lesion group

Fifteen individuals with adult-onset brain lesions that were stable and chronic (sustained more than four years beforehand) were recruited from the Cambridge Cognitive Neuroscience Research Panel at the MRC Cognition and Brain Sciences Unit, Cambridge. Individuals were selected based on lesion location in the ventromedial prefrontal cortex (vmPFC; n = 8) or lateral prefrontal cortex (latPFC; n = 7).

**Fig. 1:**
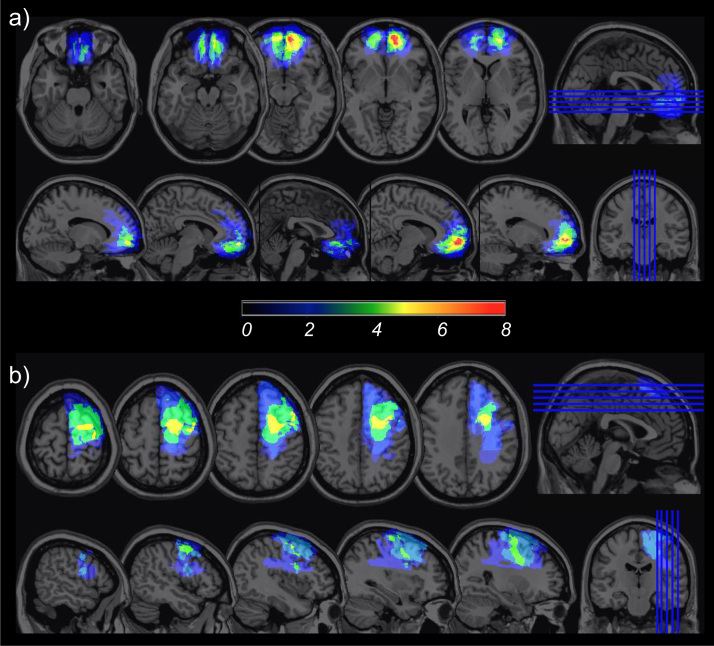
Lesion overlap for (a) vmPFC group and (b) latPFC group. Colour bar indicates the number of individuals with lesions overlapping at each voxel. (For interpretation of the references to color in this figure legend, the reader is referred to the web version of this article.)

Lesion location and overlap is shown in Fig. 1. All individuals underwent MRI with a 1.5-T or 3-T scanner. MRIcro software (Rorden and Brett, 2000) was used for manual lesion tracing, volume calculation and visualisation of lesion overlap. Images were normalised to the Montreal Neurological Institute (MNI) standard brain using SPM99 (Statistical Parametric Mapping; Wellcome Department of Cognitive Neurology, London, UK). The vmPFC group included individuals with the following lesion aetiologies: meningioma resection (n = 5), anterior communicating artery aneurysm (n = 2), and subarachnoid haemorrhage (n = 1). This group sustained damage to one or more of the following regions of the vmPFC: gyrus rectus/orbital gyrus, medial superior frontal gyrus, orbital aspect of the superior frontal gyrus, pregenual/subgenual anterior cingulate cortex. Although some lesions extended dorsally to involve the superior or middle frontal gyri or anterior cingulate, or extended more laterally in the medial frontal cortex, all lesions overlapped in the vmPFC. The latPFC group included individuals with the following lesion aetiologies: meningioma resection (n = 5), arteriovenous malformation stereotactic radiosurgery (n = 1), and ischemic stroke (n = 1). This group sustained damage overlapping in the lateral, superior region of the PFC, involving the middle frontal, inferior frontal or precentral gyri, with some lesions also extending more medially into the superior frontal gyrus, or posteriorly into parietal lobe. The two patient groups did not differ in total lesion volume (vmPFC group: mean = 38 353 mm^3^, latPFC group: mean = 58 539 mm^3^, *t*(8.61) = 0.8, *p =* 0.45) and there was no overlap in lesions between the two groups.

The lesion groups were matched for duration since lesion onset [vmPFC: 12.63±4.53 years; latPFC: 15.00±5.54 years; *t*(11.66) = 0.90, *p* = 0.39], premorbid intelligence as assessed by the National Adult Reading Test (NART) [NART predicted full scale IQ vmPFC: 114.00±7.17; latPFC: 117.71±5.06; *t*(12.51) = 1.17, *p* = 0.27], and for their performance on a measure of fluid reasoning ability, the Cattell Culture Fair Intelligence Test (Scale 2) [Cattell scores vmPFC: 29.88±8.54; latPFC: 31.57±6.53; *t*(12.81) = 0.43, *p* = 0.67].

#### 2.1.2 Control groups

Seventeen healthy subjects (age range: 52–71 years) were selected from a volunteer panel to serve as age-matched controls for the lesion groups. To replicate the findings from this first older control group and contrast them with a younger control group, we recruited a further 15 age-matched older adults (age range: 55–76 years), as well as 15 young adults (18–25 years). Control participants were excluded if they had a prior history of cognitive decline, psychiatric illness, significant head injury, movement disorders, cerebrovascular disease, alcohol or other drug abuse, or limited English proficiency. Ages of the five participant groups are shown in Table 1. There was no significant age difference between the lesion groups or the two older control groups [F (3,43) = 0.40, *p* = 0.75].

**Table 1:** Overview of participant groups.

**Group**	**N**	**Age, years (SD)**	**Sex (M/F)**
**vmPFC**	8	62.13 (10.22)	4/4
**latPFC**	7	62.43 (6.48)	3/4
**Age-matched healthy control group**	17	63.06 (6.23)	11/6
**Older control group**	15	65.07 (6.86)	6/9
**Younger control group**	15	21.20 (2.11)	5/10

vmPFC = ventromedial prefrontal cortex; latPFC = lateral prefrontal cortex.

All participants in the study provided informed consent in accordance with the Declaration of Helsinki and the study was approved by the local ethics committee. Participants were reimbursed for their time at standard rates and they were not paid in accordance with their performance on the task.

### 2.2 Contingency learning task

#### 2.2.1 Contingency manipulation

We employed a novel contingency learning task (Vaghi et al., 2018). The task was designed to measure behavioural output and subjective evaluation in response to changes in two conditional probabilities: i) the probability of an outcome given an action, P(O|A), i.e., the probability of a response-contingent outcome; and ii) the probability of an outcome given the absence of an action, P(O|~A), i.e., the probability of a non-contingent outcome. The difference between these two probabilities is represented by ΔP, where ΔP˭P(O|A)–P(O|~A) (Hammond, 1980).

In the task, P(O|A) and P(O|~A) were varied within each block so that participants experienced different levels of contingency (outcome-response relationship). There were two levels of *positive instrumental contingency*, in which P(O|A) was higher than P(O|~A), so that performing an action increased the probability of reward. There were also two levels of instrumental *negative contingency*, in which P(O|~A) was higher than P(O|A), so that performing an action decreased the probability of a rewarding outcome. *Zero contingency* occurred when P(O|A) and P(O|~A) were equal, such that the action had no causal effect on the outcome.

The specific probabilities and contingencies are shown in Table 2. The task includes block transitions in which P(O|A) and P(O|~A) change in isolation (with the other held constant at zero), and others in which P(O|A) and P(O|~A) change simultaneously (i.e., both conditional probabilities differed from zero). We wished to examine the effects of altering P(O|A) and P(O|~A) separately, to test the hypotheses that the vmPFC parametrically tracks the value of P(O|A) (Liljeholm et al., 2011) and that ageing has specific effects on sensitivity to changes in P(O|~A) (Mutter and Williams, 2004). We therefore examined only blocks in which a single probability had changed and the other was held constant at zero, resulting in the conditions described in Table 2, where P(O|A) and P(O|~A) range from 0.0 to 0.6.

**Table 2:** Task blocks analysed where either P(O|A) or P(O|~A) were varied individually.

	Programmed contingencies				
P(O|A)	0.00	0.00	0.00	0.30	0.60
P(O|~A)	0.60	0.30	0.00	0.00	0.00
**ΔP**	**−0.60**	**−0.30**	**0.00**	**0.30**	**0.60**

Probabilities (P) of an outcome (O) given an action (A) or its absence (~A): the response-contingent conditional probability P(O|A), the response-non-contingent conditional probability P(O|~A), and the instrumental contingency i.e., the difference between these two, where ΔP˭P(O|A)–P(O|~A).

#### 2.2.2 Task structure

The task was programmed in MATLAB (MathWorks) using Psychtoolbox 3. Participants were instructed they would see a white triangle on the screen, which they could respond to by pressing the space bar, or they could choose not to press. They were informed that pressing could earn them a 25 pence reward; however, the relationship between pressing and obtaining a reward might vary, such that pressing may not always result in a reward, or it might prevent a reward, or a reward might arrive on its own. Participants were encouraged to press or not press, depending on which one was associated with a favourable outcome, and told that the goal of the task was to maximise the amount of money won.

There were 12 blocks, each lasting 60 s. The first 3 training blocks (high contingency, degraded contingency and zero contingency) were presented in the same order, providing an implicit learning phase. The remaining 9 blocks were presented in a Latin square design across participants and the contingency differed across blocks according to variations in P(O|A) and P(O|~A). The block order and programmed contingencies are shown in Supplementary Table 1. After each block, subjective evaluation of the response-outcome contingency was obtained; participants were asked to rate the extent to which pressing the space bar caused a reward or prevented a reward. Ratings were registered using a visual analogue scale that ranged from −100 to 100, corresponding to the space bar always preventing (−100) or always causing (100) a reward, with the zero midpoint indicating that pressing the space bar had no effect on reward delivery.

Given that responses were free-operant and self-paced, the task was structured so that participants would experience similar reward delivery rates regardless of their pressing rate. This ensured each participant experienced the intended programmed contingency in each block (see Supplementary Table 1). To achieve this, blocks were divided into 1-second bins (Hammond, 1980). If a response occurred during a bin, the triangle would turn yellow and the outcome was delivered at the end of the bin according to the defined P(O|A) for that block. If no response occurred the outcome was delivered according to the P(O|~A) for that block. When the outcome was a reward, an image of 25 pence, together with a tone and the text “Reward, you win!”, was displayed at the end of the bin for 500 ms. If the bin was unrewarded, no feedback occurred. Only the first space bar press within a bin was recorded and had programmed consequences. A running total of money accumulated was displayed on the top right hand corner of the screen. The bins went unsignalled with no inter-trial interval, so the participant did not experience these as trials per se, but they experienced each block as unstructured and their responses as free operant.

The outcome measures from the task were response rates and subjective causal judgements for the blocks of interest (i.e., those experimental blocks where P(O|A) and P(O|~A) were not varied simultaneously). Response rates were computed by dividing the number of responses by the number of bins for each block (given that blocks were divided into 1-second bins, the rate would equate to responses per second). To identify distinct contributions of vmPFC damage or ageing, we conducted separate analyses across those blocks where P(O|A) was varied and P(O|~A) was held at zero (i.e., for positive instrumental programmed contingencies 0, 0.3, and 0.6), versus those blocks where P(O|~A) was varied and P(O|A) was held at zero (i.e., for negative instrumental programmed contingencies 0,−0.3, and −0.6).

We did not explicitly examine contingency degradation using in this study, i.e., the transition from a positive contingency to a zero/low contingency by increasing P(O|~A). Given the Latin Square design, these transitional probabilities were not fixed. However, the training blocks, Block 1 (positive contingency of 0.6) vs. Block 2 (zero contingency), do satisfy conditions of contingency degradation. Results from these blocks must be interpreted cautiously, as they were considered training blocks to familiarise participants with the task. However, to verify that the groups were sensitive to contingency degradation, we conducted a post hoc analysis on those blocks. This is reported in Supplementary Material. Together, this analysis suggested all groups were able to modify their responding following contingency degradation. Both the lesion groups and younger controls showed less response adaptation following contingency degradation, relative to older controls.

### 2.3 Statistical analysis

Welch's Independent samples *t*-tests and ANOVAs with Tukey *post hoc* tests were used to compare demographics and background clinical measures. For the contingency learning task analyses were performed in R version 3.3.1 (http://www.r-project.org/) using the ‘ez’ package for ANOVA (Lawrence, 2016). Levene's test was used to verify homogeneity of variance. Mauchly's test of sphericity was applied and Greenhouse–Geisser or Huynh–Feldt corrections were employed for ε < 0.75 or ε ≥ 0.75, respectively. Performance on the contingency learning task was analysed using linear mixed-effects models. Group was used as a fixed-effect factor. The maximal random effect structure justified by the design was specified (Barr et al., 2013). Analysis scripts and data from the contingency learning task are available here https://doi.org/10.17863/CAM.17553 via the University of Cambridge Data Repository.

## 3 Results

### 3.1 Response output in lesion groups and older controls

#### 3.1.1 Response rates in the vmPFC lesion group show adaptation to contingency changes

**Fig. 2:**
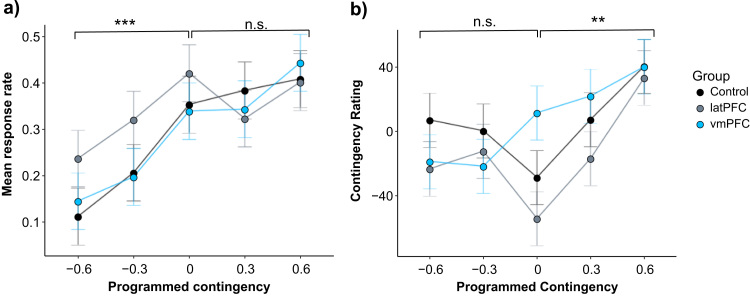
Response rates and subjective contingency ratings across levels of programmed contingency for the lesion groups and their control group. a) Response rates show an overall tendency to increase with instrumental contingency. This effect was significant for negative instrumental contingencies, with all groups showing significant differences across levels of programmed contingency [denoted by ***], but significant differences were not observed for the positive instrumental contingencies. b) When the instrumental contingency was positive, contingency ratings showed the anticipated increase across levels of increasing programmed contingency. However, only the controls and latPFC groups showed significant differences in their ratings across levels of programmed contingency [denoted by **] suggesting that the vmPFC group was less sensitive to changes across these levels. In contrast, for changes in negative instrumental contingencies indicated by increasing non-contingent outcome probabilities (−0.6, −0.3, 0) the expected increase in ratings was not apparent in any of the three groups. Error bars show Fisher's Least Significant Difference (FLSD) to facilitate post-hoc within-subjects comparisons (error bars are ± 0.5 × t_critical_ x SD); n.s. = not significant.

As the non-contingent relationship changed, (across programmed contingencies 0, −0.3, and −0.6), all groups responded more as contingency increased (Fig. 2a) [F(2,58) = 18.58, *p* < 0.000001, generalised η^2^ = 0.15]. There was no main effect of group [F(2,29) = 0.76, *p* = 0.48, generalised η^2^ = 0.04] and the group × contingency interaction was not significant [F(4,58) = 0.17, *p* = 0.95, generalised η^2^ < 0.01], suggesting that the groups showed similar modulation of their response rates across levels of negatively correlated instrumental response contingent relationships. All groups showed significantly higher mean response rates at 0 compared to −0.6 (FLSD test). Controls and vmPFC showed a significant increase in response rates at a contingency of −0.3 versus 0.

In contrast, the effect of contingency upon responding was weaker for positively correlated response-contingent relationships (across instrumental programmed contingencies 0, 0.3, and 0.6). There were no main effects for programmed contingency or group, and the interaction was not significant [programmed contingency main effect: F(2,58) = 1.83, *p* = 0.17, generalised η^2^ = 0.01; group main effect: F(2,29) = 0.01, *p* = 1.00, generalised η^2^ < 0.001; interaction: F(4,58) = 0.86, *p* = 0.48, generalised η^2^ = 0.01]. Despite controls and vmPFC having higher mean response rates at 0.6 compared to 0, these effects were not significant.

### 3.2 Awareness of causality in lesion groups and older controls

#### 3.2.1 vmPFC patients show reduced sensitivity to detecting changes in response-outcome associations

Causal ratings increased as a function of positive instrumental programmed contingency across the 0, 0.3 and 0.6 levels (Fig. 2b) [F(2,58) = 19.17, *p* < 0.000001, generalised η^2^ = 0.30]. There was a main effect of group [F(2,29) = 4.12, *p* < 0.01, generalised η^2^ = 0.10], with no contingency × group interaction [F(2,58) = 1.12, *p* = 0.361, generalised η^2^ = 0.05]. The group effect was driven by overall higher ratings in the vmPFC group compared to the control and latPFC groups, as shown by post-hoc *t*-tests [vmPFC vs. control: *t*(57.767) = −1.7037, *p* = 0.09; vmPFC vs. latPFC: *t*(36.043) = −2.6292, *p* < 0.05].

Subjective ratings of control and latPFC groups showed clear sensitivity to positive contingencies, but those of the vmPFC group did not. Post-hoc within-subject comparisons using FLSD revealed that controls showed significant differences in their detection rates across the three neighbouring levels 0, 0.3 and 0.6. The latPFC group also showed significant differences in their detection rates between the neighbouring 0.3 and 0.6 conditions, and their ratings at 0.6 were significantly higher than at 0. Together, these results suggest that causality judgements in the control and latPFC groups were sensitive to changes in contingency. In contrast, the vmPFC group showed no differences in detection rates across neighbouring conditions or between 0 and 0.6, suggesting reduced awareness of contingency changes when P(O|A) was varied. However, these group differences must be interpreted in the context of a lack of significant contingency × group interaction.

#### 3.2.2 Lesion groups and age-matched controls both showed impaired awareness of changes in non-contingent associations

Both the lesion groups and the age-matched controls showed a lack of sensitivity to negative contingencies in their subjective judgements of causality. As shown in Fig. 2b, causal ratings did not consistently increase as a function of changes in P(O|~A) across the −0.6, −0.3, and 0 levels, with no main effect of programmed contingency [F(2,58) = 2.12, *p* = 0.15, generalised η^2^ = 0.04]. There was no group difference in overall ratings [F(2,29) = 1.87, *p* = 0.17, generalised η^2^ = 0.05]; however the contingency × group interaction was significant [F(4,58) = 2.97, *p* < 0.05, generalised η^2^ = 0.12]. Follow-up tests of simple effects revealed a group difference in ratings at the 0 contingency level [F(2,29) = 4.02, *p* < 0.05, generalised η^2^ = 0.22].

Neither lesion group showed any differences in ratings across these contingency levels (FLSD post-hoc test). Controls had significantly lower ratings for the ΔP = 0 condition compared to ΔP = −0.3, which is the opposite of what would be expected based on programmed contingency. Together, these results suggest that all groups showed impaired awareness of causality when non-contingent probabilities were varied, despite being able to adapt their response rates to these variations.

### 3.3 Response output in younger versus older controls

To explore whether the impairment in rating negative contingencies was related to age, we recruited an independent group of older adult participants (n = 15), who were matched for age to the lesion control group. We contrasted this new control group with a group of young adults (n = 15). (Note, this second group of older controls showed significant differences in their responses to positive contingency, in contrast to the controls in the lesion group comparison. We therefore conducted a post hoc analysis comparing the lesion groups against the entire cohort of combined older controls, N = 32. See Supplementary material for details).

#### 3.3.1 Younger and older adults do not differ in response rates

**Fig. 3:**
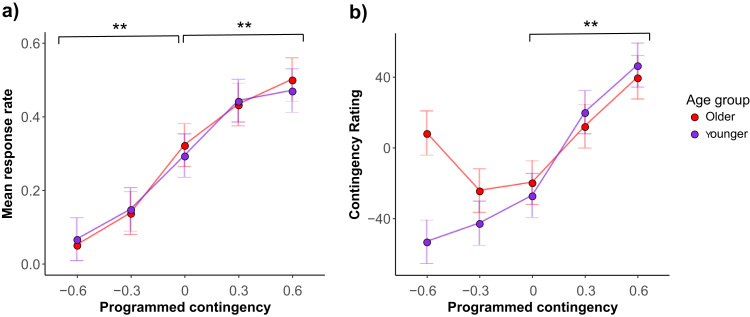
Response rates and subjective contingency ratings across levels of programmed contingency for younger and older adults. a) Response rates show an overall tendency to increase with the increase of instrumental contingency. This effect was significant for both negative and positive instrumental contingencies, with both groups showing significant differences across levels of programmed contingency [denoted by **]. b) Contingency ratings in both groups show the anticipated increase as the probability of response-contingent outcome increased (programmed contingency levels 0,0.3,0.6) [denoted by **]. In contrast for changes in non-contingent outcome probabilities (−0.6, −0.3, 0), younger adults show the expected increases in their ratings, but this is absent in the older adults. Error bars show Fisher's Least Significant Difference (FLSD).

Both younger and older adults showed a robust and identical effect of contingency upon responding. Fig. 3a shows that for the changes in non-contingent relationships (i.e., programmed contingencies 0, −0.3, and −0.6), response rates for the older and younger groups increased as a function of programmed contingency [F(2,56) = 6.00, *p* < 0.00001, generalised η^2^ = 0.31]. There was no main effect of group [F(1,28) = 0.0008, *p* = 0.98, generalised η^2^ < 0.01] and the group by programmed contingency interaction was not significant [F(2,56) = 0.24, *p* = 0.71, generalised η^2^ < 0.01], suggesting that the groups showed similar modulation of their response rate according to instrumental contingency. Within group comparisons using FLSD confirmed that both groups showed significantly different response rates between the −0.6 and 0, and between the −0.3 and 0 conditions.

Similarly for changes in response-contingent relationships (i.e., instrumental programmed contingencies 0, 0.3, and 0.6), response rates for the older and younger groups increased as a function of programmed contingency [main effect: F(2,56) = 10.11, *p* < 0.001, generalised η^2^ = 0.08]. There was no main effect of group [F(1,28) = 0.04, *p* = 0.84, generalised η^2^ < 0.01] and the group by programmed contingency interaction was not significant [F(2,56) = 0.16, *p* = 0.87, generalised η^2^ < 0.01]. Within group comparisons using FLSD confirmed that both groups showed significantly increased response rates between the 0 and 0.6 conditions, and between the neighbouring conditions 0 and 0.3.

### 3.4 Awareness of causality in younger and older controls

#### 3.4.1 Younger and older adults do not differ in their causal judgements for changes in positive response-outcome relationships

Both younger and older adults showed robust and similar effects of contingency upon causality judgements for positive contingencies, but the groups’ judgements differed for negative contingencies. As illustrated in Fig. 3b, contingency ratings across levels 0, 0.3 and 0.6 increased as a function of programmed contingency, as evidenced by a main effect [F(2,56) = 38.04, *p* < 0.00000001, generalised η^2^ = 0.40]. There was no main effect of group [F(1,28) = 0.07, *p* = 0.80, generalised η^2^ < 0.01] and the group × contingency interaction was not significant [F(2,56) = 0.62, *p* = 0.50, generalised η^2^ < 0.01], suggesting that the groups showed similar awareness of causality across these levels of programmed contingencies. Within-group comparisons using FLSD confirmed that both groups showed significantly larger mean differences between each pair of neighbouring contingencies.

#### 3.4.2 Older adults showed impaired awareness of changes in non-contingent associations

Older adults made impaired causality judgements compared to younger adults for non-contingent conditional probabilities. As illustrated in Fig. 3b, younger adults showed the expected increase in causality ratings across levels −0.6, 0.3 and 0, but older adults did not consistently increase their ratings. This was reflected in a significant group × contingency interaction [F(2,56) = 4.14, *p* = 0.04, generalised η^2^ = 0.07]. There was a significant main effect of group upon contingency rating [F(1,28) = 7.10, *p* < 0.05, generalised η^2^ = 0.11], but no significant effect of programmed contingency [F(2,56) = 0.76, *p* = 0.43, generalised η^2^ = 0.01].

Older adults showed higher subjective ratings that erroneously decreased with increasing contingencies, whereas younger adults showed the anticipated increase in their subjective ratings as a function of changes in P(O|~A). The older group had significantly higher ratings overall [post hoc *t*(87.81) = 3.12, *p* < 0.01]. Group ratings differed significantly at the −0.6 level [simple effect, F(1,28) = 19.23, *p* < 0.001, generalised η^2^ = 0.41]. Despite higher mean ratings at each level in the younger controls, these did not differ significantly when compared by FLSD. In contrast, in older adults higher causality ratings were observed for extremely negative contingency (ΔP = −0.6) compared with lower levels of negative contingency (ΔP = −0.3), which is the opposite of what would be predicted based on programmed contingency.

## 4 Discussion

Our findings suggest that vmPFC lesions are not associated with deleterious effects on instrumental response rates, but they are linked to impaired causal awareness of contingencies between actions and outcomes. Similarly, in healthy ageing, we observed intact response rates, but awareness of causality was impaired for non-contingent probabilities. These results highlight two contexts where performance and awareness have been dissociated. We have thus shown that learning response-outcome contingencies to guide behaviour can occur while subjective awareness of those contingencies is impaired. Our findings suggest that prefrontal damage and ageing both impair mechanisms that support the awareness of causal relationships acquired during learning.

Individuals with vmPFC lesions responded to contingency changes in a way similar to age-matched controls (Fig. 2a). They showed intact ability to modulate their response rates across several levels of instrumental negative contingency. Consistent with the pattern of performance observed in controls, the vmPFC group showed (non-significant) modulation of their response rates across levels of instrumental positive contingency. In contrast, the vmPFC group showed reduced sensitivity in their causal awareness when compared to their respective controls and the latPFC group. This reduced sensitivity was apparent for awareness of positive contingencies, where, in the absence of a clear contingency × group interaction, post hoc tests identified reduced adaption to contingency changes in the vmPFC group. All groups were impaired in their awareness of negative contingencies, which is discussed later in the context of a general ageing effect.

The vmPFC has an established role in computing action-outcome contingencies. However, our finding of preserved response rates in the vmPFC lesion group emphasises that this information must also be signalled in other brain regions. Other neural substrates involved in learning action-outcome contingencies include the anterior and posterior caudate (Tanaka et al., 2008, Liljeholm et al., 2011). Furthermore, integrated information processing of contingent and non-contingent action-outcome associations (and also action-reward contingencies) occurs across diverse cortical regions, including the dorsolateral prefrontal cortex and inferior and superior parietal lobules (Seo et al., 2007, Seo et al., 2009, Liljeholm et al., 2011). These areas encode a conjoint history of action-outcome associations, which may support aspects of associative learning in the face of medial prefrontal lesions (Walton et al., 2010). However, to our knowledge, the possibility that individuals with chronic vmPFC lesions may show a greater reliance on dorsolateral or posterior parietal regions to signal contingent probabilities is yet to be directly tested. Of further consideration, is the possibility that preserved responding in the vmPFC group was supported by an intact habit system (i.e., including the posterior putamen). However, it is unlikely that responding to negative contingencies was strongly mediated by the habit system in our vmPFC group, as their clear sensitivity to non-contingent manipulations is suggestive of goal-directed behaviour. Their responses to positive contingencies were less sensitive to contingency changes, which could imply a more habitual response style, possibly consistent with transference to intact habit circuitry.

Our results fit with a recent study where individuals with vmPFC lesions (n = 6) demonstrated intact acquisition of instrumental reward contingencies (Reber et al., 2017). In that study, vmPFC patients displayed normal learning of stimulus-response associations to earn food rewards (i.e., they learned two key presses that led to two different foods). The stimulus-response associations were fixed, suggesting that the vmPFC is not critical for relatively simple stimulus-response learning. The authors hypothesised that intact habit-based brain systems may instead be supporting learning in this context. Preserved ability to establish response-outcome associations was also recently shown in individuals with lesions to the vmPFC, as their choices during a three-armed bandit task revealed intact credit assignment (i.e., they could correctly attribute outcomes to a precipitating stimulus choice) (Noonan et al., 2017). We extend this notion to a more dynamic form of learning, in which response-outcome associations were not fixed. Our findings suggest that the vmPFC may not be critically involved in monitoring ongoing fluctuations in response-outcome contingencies, and that this behaviour can be supported via information from other cortical areas and the caudate. Interestingly, in the study by Reber and colleagues, following successful learning, the vmPFC group was subsequently impaired on a measure of outcome devaluation; they failed to reduce responding for food rewards that were devalued by feeding to satiety. Taken together with our results, this suggests an important dissociation: the vmPFC may be critically involved in updating behaviour based on the current incentive value of reward (cf. impaired outcome devaluation), but it is not critical for updating behaviour based on changes in the statistical frequency of reward occurrence (cf. intact contingency learning).

Similar dissociations have been reported in monkey and rodent work. OFC lesioned monkeys showed intact contingent and non-contingent learning, but were impaired in updating stimulus-outcome associations based on their current biological value, or “desirability” (Rudebeck et al., 2017). Medial orbitofrontal lesions in the rat impaired outcome devaluation, but left contingency learning intact (as measured by sensitivity to contingency degradation) (Bradfield et al., 2015). If we assume cautiously that the large vmPFC lesions we report here subsume the homologue of the rat medial orbital cortex, our results provide further evidence that encoding action-outcome contingencies is not dependent on this region. In the series of experiments by Bradfield et al., the lesioned animals were impaired on tasks requiring retrieval of previously learnt associations (e.g., outcome devaluation and Pavlovian-instrumental transfer), but were unimpaired on tasks where the outcomes were readily observable, including contingency learning. This may speak to the role of the medial orbitofrontal cortex in encoding an overarching “cognitive map” of the task at hand, integrating the history of diverse learning and sensory signals relevant to form a detailed model of the task structure (Rudebeck and Murray, 2014; Wilson et al., 2014; Stalnaker et al., 2015; Rolls, 2017 this issue), thus allowing an agent to make inferences about outcomes that are not readily observable. This is consistent with a role for the medial orbitofrontal cortex in supporting model-based learning (McDannald et al., 2012).

The reduced sensitivity for causal ratings we found in the vmPFC group highlights a critical role for this region in awareness. In keeping with a putative role for the medial orbitofrontal cortex in encoding an overarching model of the task environment (Rudebeck and Murray, 2014; Wilson et al., 2014, Bradfield et al., 2015; Stalnaker et al., 2015), it may be that individuals with vmPFC damage do not form this integrated model, which prevents higher-level awareness of task rules and contingences such that they can be consciously described later. More broadly, assessing convergence between objective performance, and subjective appraisal of that performance, is a means of quantifying metacognitive accuracy (Weiskrantz, 1998, Fleming and Dolan, 2012, Maniscalco and Lau, 2012). Individuals with lesions to the anterior PFC manifest impaired subjective ratings on visuo-perceptual tasks, such that their subjective awareness is not proportionate to their actual task performance (Del Cul et al., 2009, Fleming et al., 2014). Our findings identify a causal contribution of the vmPFC in the subjective awareness of response-outcome contingencies. The importance of the PFC in awareness is underscored by reports that metacognitive ability in healthy people correlates with anterior PFC volume and microstructure (Allen et al., 2017, Fleming et al., 2010).

We have also shown a striking dissociation in healthy ageing, with preserved response adaptation to contingency but impaired causal awareness for negative contingencies. In our comparison of younger and older healthy adults, response rates between the two groups were similar, and both showed a consistent increase in responding as the strength of response-outcome associations increased (Fig. 3a), though it should be noted that there was some variance between our two older control groups. Those in the lesion comparison group (n = 17) showed a blunted increase in response rates across positive contingencies. In contrast, the second group of older adults (n = 15) showed significant modulation of response rates across positive and negative contingencies. However, when combined in a post hoc analysis, the entire group of N = 32 older controls showed a stronger effect of response adaptation to positive contingency. This suggests that overall the task was sensitive to positive contingencies, despite variation in the older adults’ performance.

Despite this context of preserved response rates, both older groups showed a specific impairment in causal awareness of negative contingency. Here, we replicate a previous study that reported deficits in subjective awareness of negative contingencies in older vs. younger adults (Mutter and Williams, 2004). Our findings do not imply that ageing is associated with an overarching deficit in causal awareness or metacognition per se, as older adults’ subjective judgements were well maintained for positive contingencies. Instead, their restricted deficit for awareness of negative contingency could be interpreted within a more general framework of diminished prefrontal function in ageing (Nyberg et al., 2010). Ageing is associated with a decline in fluid executive abilities, including working memory, attention and task switching, as well as psychomotor slowing (Grady, 2012). Of specific relevance to our findings, older adults exhibit greater dual-task costs compared to younger adults (Verhaeghen et al., 2003).

The ability to learn action-outcome contingencies and execute them behaviourally, whilst simultaneously monitoring performance to the extent it can be accurately evaluated post hoc, may be a form of dual-tasking. If so, it could be predicted that awareness would be compromised in situations where maintaining task performance is increasingly challenging. This hypothesis was recently tested with respect to metacognitive awareness of visuo-perceptual performance. Subjects’ metacognitive sensitivity was impaired when they were required to perform a concurrent working memory task, during their performance of the visual discrimination task that they would subsequently appraise (Maniscalco and Lau, 2015). This indicates that metacognition, or awareness, may suffer a performance cost when additional cognitive resources are devoted to maintaining behavioural output. In our case, this would suggest that, for older adults, it is a relatively more challenging task to learn and respond to changes in negative contingency. Older adults show learning deficits relative to younger adults in environments that are especially dynamic or uncertain, or when feedback is ambiguous (Eppinger et al., 2011, Eppinger and Kray, 2011, Pietschmann et al., 2011, Nassar et al., 2016). Under conditions of negative contingency, performing an action reduces the probability of a reinforcing outcome. This conceivably entails a counterintuitive task structure, which may be associated with elevated levels of uncertainty. Although older adults successfully adapt their responding to negative contingency, this may be a more demanding task for them, consequently limiting cognitive resources available for metacognitive processing.

We have presented evidence for a dissociation between responding to changes in action-outcome contingencies, and being subjectively aware of those contingencies. We showed that participants could achieve successful behavioural adaptation to variations in action-outcome contingencies, without showing the same degree of subjective awareness. Our findings suggest, anatomically, that the vmPFC may be critical for supporting subjective awareness of causality. In addition, we have advanced the hypothesis that impaired causal awareness in ageing may result from costs in dual-tasking performance. More broadly, we have highlighted contingency learning as a context where performance and awareness can dissociate – complementing those seminal reports of performance-awareness dissociations first revealed by blindsight in posterior cortical regions and by preservation of priming and conditioning in amnestics (Warrington and Weiskrantz, 1968, Weiskrantz and Warrington, 1979, Weiskrantz, 1986, Weiskrantz, 2004).
