# Supplementary material for: Impaired awareness of action-outcome contingency and causality during healthy ageing and following ventromedial prefrontal cortex lesions
Source: Neuropsychologia. 2019 May;128:282–9. doi: 10.1016/j.neuropsychologia.2018.01.021 (PMC6562272; doi:10.1016/j.neuropsychologia.2018.01.021)
Supplement: Supplementary file 1 — Supplementary material [file mmc1.pdf]

Supplementary material - Impaired awareness of action-outcome contingency and causality during healthy ageing and following ventromedial prefrontal cortex lesions

Supplementary Table 1. Programmed contingency levels for each block and the actual experienced contingencies for each group

		Block1	Block2	Block3	Block4	Block5	Block6	Block7	Block8	Block9	Block10	Block11	Block12
Programmed contingency	P(O|A)	0.6	0.6	0	0	0	0	0.3	0.3	0.3	0.6	0.6	0.6
	P(O|-A)	0	0.6	0	0	0.3	0.6	0	0.3	0.6	0	0.3	0.6
	DeltaP	0.6	0	0	0	-0.3	-0.6	0.3	0	-0.3	0.6	0.3	0
Experienced contingency	vmPFC	0.62	0.11	0.00	0.00	-0.29	-0.59	0.30	-0.02	-0.27	0.60	0.34	-0.06
	latPFC	0.63	0.05	0.00	0.00	-0.25	-0.67	0.28	0.03	-0.15	0.59	0.37	-0.20
	Lesion controls	0.61	0.04	0.00	0.00	-0.29	-0.59	0.31	-0.02	-0.21	0.60	0.29	-0.05
	Older controls	0.63	0.11	0.00	0.00	-0.29	-0.59	0.30	-0.04	-0.10	0.61	0.44	-0.06
	Younger controls	0.63	0.10	0.00	0.00	-0.29	-0.59	0.28	-0.04	-0.25	0.59	0.32	-0.17

Supplementary Table 1 shows the block design and programmed contingencies. The experienced contingencies are also shown, i.e., given that responses were free-operant and self-paced, the task was structured so that participants would experience similar reward delivery rates regardless of their pressing rate. This ensured each participant experienced the intended programmed contingency in each block. See over page for calculation of experience contingency

Experienced contingency

We computed mean experienced contingency for each subject per block using the formula (i) that was based on the formula for delta P [ΔP=P(O｜A)-P(O|~A)].

(i) [C1/((C1+C2))]-[C3/((C3+C4))]

Where C1 = the number of contingent outcomes (rewards delivered upon key press); C2 = the number of times that a key press was not associated with the delivery of an outcome; C3 = the number of non-contingent outcomes (rewards delivered in the absence of a key press); C4 = the number of times that there was no key press and no outcome delivered.

There was a high correlation between mean experienced contingencies and programmed contingencies for all groups: vmPFC (r = 0.994, p< 0.0000000001); latPFC (r = 0.974, p< 0.0000001); lesion controls (r = 0.996, p< 0.0000000001); older controls (r = 0.977, p< 0.00000001); younger controls (r = 0.984, p< 0.00000001). We also compared the experienced contingencies between the groups in each experiment and found no significant differences: vmPFC and latPFC lesion groups with their respective control group [F(2,33) = 0.001, p = 0.999]; older vs. younger adults $t(22)$ = -0.25, p = 0.81.

Contingency degradation

We conducted a post hoc examination of the response rates in Block 1 vs. Block 2. These blocks represent a positive contingency of 0.6 (Block 1) and a zero contingency (Block 2). These blocks therefore satisfy conditions to examine contingency degradation, i.e., when a participant is transitioned from a positive contingency to a zero/low contingency by increasing P(O|~A). We interpret these results cautiously, as Blocks 1-2 were designed as a training phase wherein participants were familiarising themselves with the task.

Together, the results of this analysis suggest all groups were able to modify their responding following contingency degradation. Both the lesion groups and younger controls showed less response adaptation relative to older controls.

Supplementary Figure 1

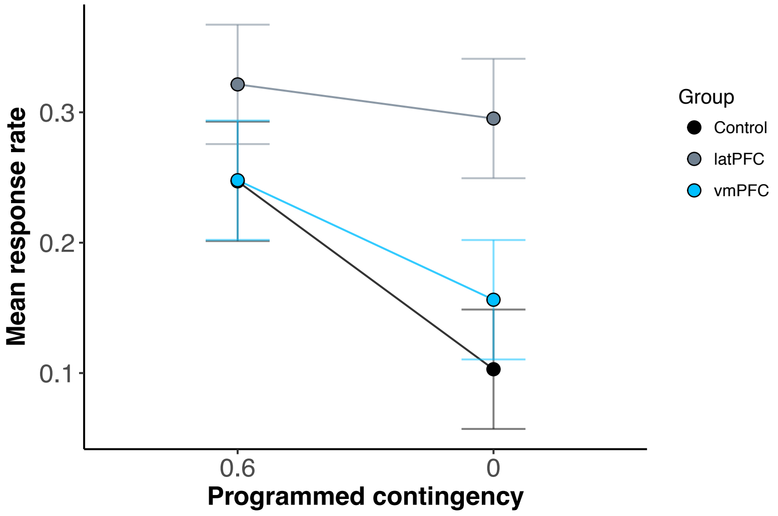

Figure shows mean response rates across the transition from high positive contingency (Block 1) to zero contingency (Block 2). Error bars show Fisher's Least Significant Difference (FLSD) to facilitate post-hoc within-subjects comparisons (error bars are ± 0.5 x $t_{critical}$ x SD)

Lesion groups and age matched controls

As the contingency was degraded (Block 1 vs. Block 2) all groups responded less [$F(1,29) = 16.56$, $p < 0.001$, generalised $\eta^2 = 0.09$]. There was no main effect of group [$F(2,29) = 1.80$, $p = 0.18$, generalised $\eta^2 = 0.09$] and the group × contingency interaction was not significant [$F(2,29) = 1.67$, $p = 0.21$, generalised $\eta^2 = 0.02$]. These results suggest a similar profile of responding across all three groups in response to

the transition from positive to zero contingency. Although, it should be noted that Supplementary Figure 1 shows that response adaptation was relatively minimal in the lateral PFC group. Post hoc FLSD tests revealed that only controls showed a significant decrease in response rates between the 0.6 and zero contingencies.

Supplementary Figure 2

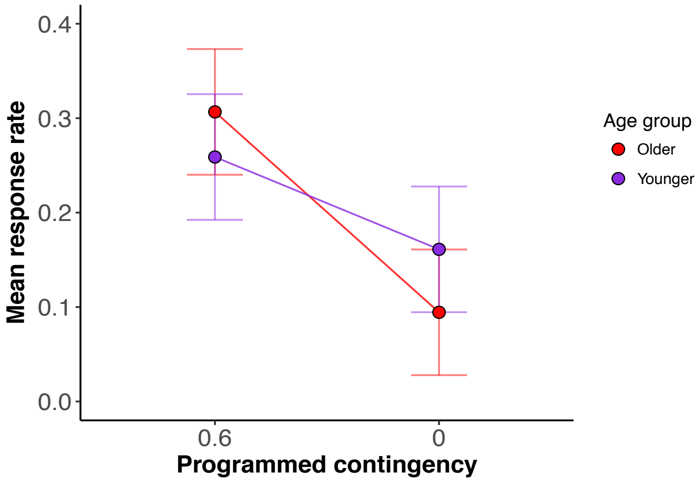

Figure shows mean response rates across the transition from high positive contingency (Block 1) to zero contingency (Block 2). Error bars show Fisher's Least Significant Difference (FLSD) to facilitate post-hoc within-subjects comparisons (error bars are ± 0.5 x $t_{critical}$ x SD)

Older and younger groups

As the contingency was degraded (Block 1 vs. Block 2) both groups reduced their responding [$F(1,28) = 11.37$, $p < 0.01$, generalised $\eta^2 = 0.10$]. There was no main effect of group [$F(1,28) = 0.02$, $p = 0.90$, generalised $\eta^2 < 0.001$] and the group × contingency interaction was not significant [$F(1,28) = 1.55$, $p = 0.22$, generalised $\eta^2 = 0.02$]. Post hoc FLSD tests revealed that between the 0.6 and zero contingencies, only the older group showed a significant decrease in their responding.

Analysis of response rates in combined older control groups

Given the variability in response to positive contingency across the two experiments, we conducted a post hoc analysis combining the older controls from both experiments, yielding N= 32. This combined older control group was analysed against the lesion groups, to determine if the larger N might help clarify interpretation of performance in the vmPFC group.

To summarise, our results show that with the larger N, older controls showed statistically stronger effects of both positive and negative contingencies. With respect to the vmPFC group, the post hoc analysis suggests that their response to positive contingencies was less robust than controls when the combined group was examined.

Response output in lesion groups and combined older controls

As the non-contingent relationship changed (across programmed contingencies 0, -0.3, and -0.6), all groups responded more as contingency increased [$F(2,88) = 36.02$, $p < 0.0000000001$, generalised $\eta^2 = 0.20$] (Supplementary Figure 3). There was no main effect of group [$F(2,44) = 1.69$, $p = 0.20$, generalised $\eta^2 = 0.05$] and the group × contingency interaction was not significant [$F(4,88) = 0.36$, $p = 0.84$, generalised $\eta^2 < 0.01$]. These results suggest that when the entire cohort of older controls was combined, the groups showed similar modulation of their response rates across levels of negatively correlated instrumental response contingent relationships. This was a similar pattern observed in the lesion vs. control analysis reported in the main manuscript (cf. Figure 2a). Similar to the original analysis, all groups showed significantly higher mean response rates at 0 compared to -0.6, and controls and vmPFC showed a significant increase in response rates at a contingency of -0.3 versus 0 (FLSD test). The only difference with the initial analysis was that, here, controls also showed a significant increase between -0.3 and -0.6; therefore, the combined older controls' responses were significantly different at each level of the non-contingent probabilities. This suggests that with the combined older cohort, we see a stronger effect of negative contingency.

Similarly, in the combined older controls, a stronger effect of contingency was evident for positively correlated response-contingent relationships (across

instrumental programmed contingencies 0, 0.3, and 0.6). In contrast to the initial analysis reported in the manuscript based on N=17 (cf. Figure 2a), here there was a main effect of programmed contingency [$F(2,88) = 5.87$, $p = 0.01$, generalised $\eta^2 = 0.03$] (Supplementary Figure 3). Similar to the initial analysis, there was no main effect of group [$F(2,44) = 0.06$, $p = 0.94$, generalised $\eta^2 < 0.01$] and the group × contingency interaction was not significant [$F(4,88) = 1.39$, $p = 0.24$, generalised $\eta^2 = 0.01$]. The effect of positive contingency was apparent in controls as their responding was significantly increased at 0.6 compared to 0 (FLSD test). Despite numerically higher responding in the vmPFC group at 0.6 compared to 0, the difference was not significant. The latPFC group showed a significant decrease in responding between 0 and 0.3, which is the opposite of what was predicted based on programmed contingency.

Supplementary Figure 3

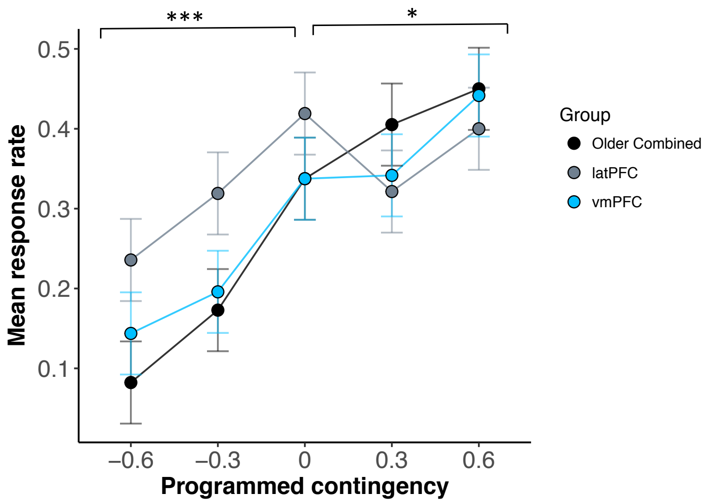

Figure shows response rates across levels of programmed contingency for the lesion groups and the combined older control group. Response rates showed an overall tendency to increase with instrumental contingency. This effect was significant for negative instrumental contingencies, with all groups showing significant differences across levels of programmed contingency [denoted by ***], but significant differences were only observed in controls for the positive instrumental contingencies 0 vs. 0.6 [denoted by *]. Error bars show Fisher's Least Significant Difference (FLSD) to facilitate post-hoc within-subjects comparisons (error bars are ± 0.5 x $t_{critical}$ x SD).
